# Identification and characterization of four immune-related signatures in keloid

**DOI:** 10.3389/fimmu.2022.942446

**Published:** 2022-07-27

**Authors:** Xiaoxiang Wang, Bo Liang, Jiehua Li, Xiaobing Pi, Peng Zhang, Xinzhu Zhou, Xiaodong Chen, Sitong Zhou, Ronghua Yang

**Affiliations:** ^1^ Guangdong Medical University, Zhanjiang, China; ^2^ Department of Burn Surgery, The First People’s Hospital of Foshan, Foshan, China; ^3^ The Second Affiliated Hospital, School of Medicine, Zhejiang University, Hangzhou, China; ^4^ Department of Dermatology, The First People’s Hospital of Foshan, Foshan, China; ^5^ Neijiang Health Vocational College, Neijiang, China; ^6^ The Second School of Medicine, Wenzhou Medical University, Wenzhou, China; ^7^ Department of Burn and Plastic Surgery, Guangzhou First People’s Hospital, South China University of Technology, Guangzhou, China

**Keywords:** immune, signature, keloid, GEO, immune infiltration

## Abstract

A keloid is a fibroproliferative disorder of unknown etiopathogenesis that requires ill-defined treatment. Existing evidence indicates that the immune system plays an important role in the occurrence and development of keloid. However, there is still a lack of research on the immune-related signatures of keloid. Here we identified immune-related signatures in keloid and explored their pathological mechanisms. Transcriptomic datasets (GSE7890, GSE92566, and GSE44270) of keloid and normal skin tissues were obtained from the Gene Expression Omnibus database. The overlap of differentially expressed genes and immune-related genes was considered as differentially expressed immune-related genes (DEIGs). Functional analysis, expression, and distribution were applied to explore the function and characteristics of DEIGs, and the expression of these DEIGs in keloid and normal skin tissues was verified by immunohistochemistry. Finally, we conducted interactive network analysis and immune infiltration analysis to determine the therapeutic potential and immune correlation. We identified four DEIGs (LGR5, PTN, JAG1, and DKK1). In these datasets, only GSE7890 met the screening criteria. In the GSE7890 dataset, DKK1 and PTN were downregulated in keloid, whereas JAG1 and LGR5 were upregulated in keloid. In addition, we obtained the same conclusion through immunohistochemistry. Functional analysis indicated that these four DEIGs were mainly involved in stem cell, cell cycle, UV response, and therapy resistance. Through interactive network analysis, we found that these DEIGs were associated with drugs currently used to treat keloid, such as hydrocortisone, androstanolone, irinotecan, oxaliplatin, BHQ-880, and lecoleucovorin. Finally, many immune cells, including CD8^+^ T cells, resting memory CD4^+^ T cells, and M1 macrophages, were obtained by immune infiltration analysis. In conclusion, we identified four immune signaling molecules associated with keloid (LGR5, PTN, JAG1, and DKK1). These immune-related signaling molecules may be important modules in the pathogenesis of keloid. Additionally, we developed novel therapeutic targets for the treatment of this challenging disease.

## Introduction

Keloid, one of the benign dermal tumors, is a pathological scar caused by abnormal hyperplasia of the connective tissue in the skin. It is a special type of skin scar; the specific formation mechanism is unclear, but it only occurs as a result of long-term abnormal wound healing (1, 2). Relevant literature shows that susceptibility to keloid formation occurs predominantly in Africans and Asians (1, 3). Keloid generally occurs between 10 and 30 years of age and affects both sexes equally. Keloid may grow slowly for weeks, months, or years, and it can gradually increase and invade the normal skin beyond the edge of the original wound, showing invasive growth, which is the most important feature of keloid (4). They eventually stop growing but do not disappear on their own. Once a keloid develops, it is permanent unless removed or treated successfully (5).

In addition to keloid, hypertrophic scar is another type of abnormal scar on the skin (6). A hypertrophic scar is a wide, thickened, and often raised scar that develops at the site of skin injury and that can be itchy and typically does not show invasive growth. Over time, it extends beyond the boundary of the scar and gradually becomes mature and flat, which is very original wound (7). A leading cause of hypertrophic scar formation is the mechanical tension on the wound (8), but a hypertrophic scar ceases to grow and spontaneously regresses without treatment ranging from months to years (6, 9), which are different from what occurs in keloid (10, 11). From an aesthetic point of view, keloid is thought of as unpleasant, and from a functional point of view, they cause pain and itching. At present, keloid treatment mainly includes surgical and non-surgical treatments, which include laser treatment, radiotherapy, pressure therapy, and hormone therapy. Surgical resection of keloid is the most direct method. Recent literature reports that fat transplantation and botulinum toxin A have certain effects on keloid treatment (12, 13). However, recurrence after conservative treatment or surgical resection is very common, causing a psychological burden on the patients (14, 15).

Inflammation is a defensive response of the human body to damage caused by various inflammatory irritants. The exudation stage includes hemodynamic changes, increased vascular permeability, and inflammatory cell response and limits. It eliminates and rejects foreign pathogenic factors and cells killed by injury through local tissue degeneration, exudation, and proliferation. After the wound is healed (usually 1 to 3 months), scars begin to proliferate on the edge of the wound, sometimes with dilated capillaries and rough surface accompanied by discomfort such as itching and pain. This stage is the collagen stage. Usually, the keloid growth reaches a peak at about 6 months, and then the keloid gradually matures and softens. The first sign of a mature keloid is a change in color, from bright red to dark red, then to purple or brown, and finally to the color of the skin around the keloid; wrinkles appear on the surface of the keloid, and capillaries are reduced until they disappear, the hardness softens, the thickness becomes thinner, and the pain symptoms eventually disappear, but the itching can persist for a long time until the keloid is fully mature (16). The maturation process of keloid is relatively slow; usually it takes half a year to 2 years, and in some patients it can take as long as 3 and 4 years, and the length of time for a keloid to mature in different parts is different (17). The treatment of a keloid needs to be comprehensively considered according to the patient’s age and its nature, size, anatomical location, and distribution as well as the existence of infection foci and function and other factors to establish an individualized treatment plan that suits a patient’s condition (18–20). A treatment plan for keloid includes an injection of corticosteroids, surgical removal (keloid surgery), pressure earring, dressing or garment, laser treatment, silicone sheets and gels, cryotherapy, radiation treatments, and ligature, but these treatments are based on appearance or symptoms. The lack of an exact treatment is mainly due to the unclear specific pathogenesis of keloid, and early literature reports showed that the levels of IgG, IgA, and IgM in keloid were higher than those in normal skin, indicating that keloid formation is a local immune response (21, 22). Moreover, the number of T cells, Langerhans cells, mast cells, and macrophages in keloid is higher than those in normal tissues, which is related to skin fibrosis (23, 24). The levels of circulating immune complexes and cells in patients with keloid are significantly higher than those in normal individuals. All the above-mentioned results show that keloid growth is closely related to immunity. Therefore, it can be inferred that immune treatment methods may have significant prospects in keloid (25).

**Figure 1:**
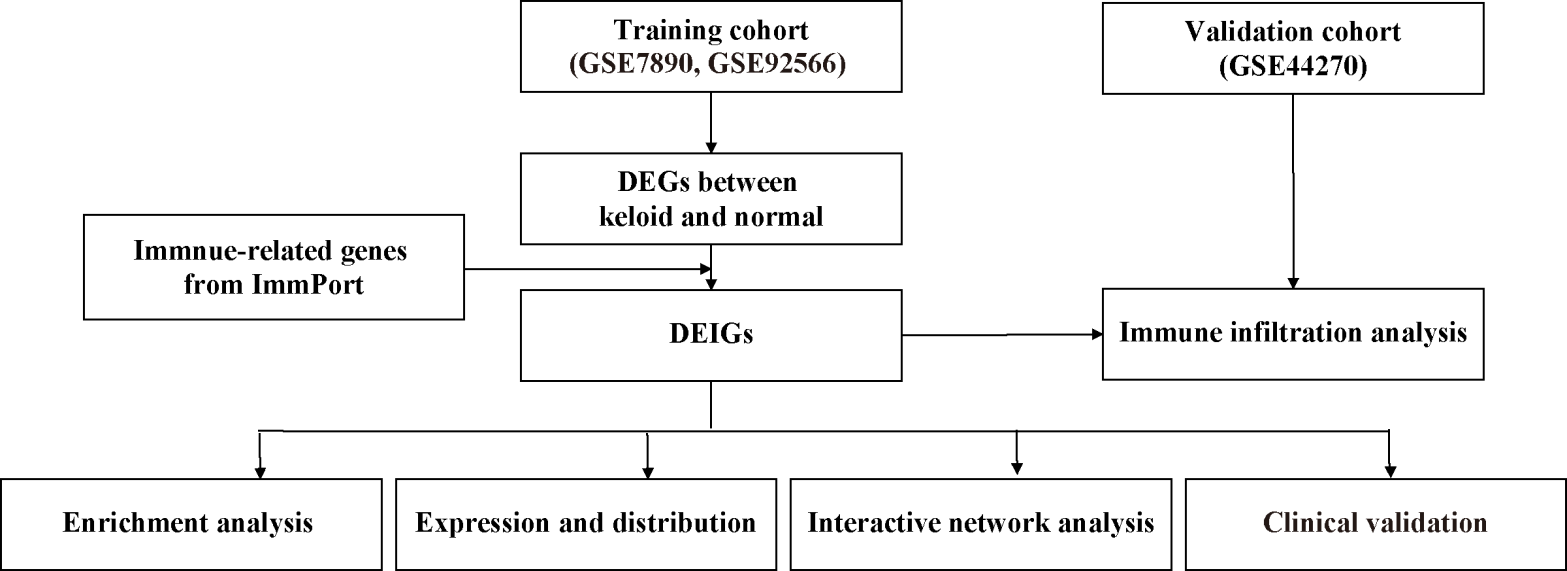
Design and workflow of this study.

Inflammation and immune responses have dramatic impacts on the pathogenesis of keloid, and there is a theory that the formation of a keloid is due to the abnormal response of fibroblasts to inflammation (11, 26). A previous study performed nine-color flow cytometry with computational analysis to detect recurrence-related epidermal cellular subsets in keloid (27), further confirming the importance of the immune system. Immune-related genes are helpful in the prognosis and mechanisms of various diseases (28–31). However, little research has been conducted on keloid. Currently, bioinformatic analysis is an important tool for analyzing expression data and screening for target genes in many diseases. Here we first explored the differentially expressed immune-related genes (DEIGs) among different keloid datasets, conducted multiple functional enrichment analyses, constructed a protein–protein interaction (PPI) network, and explored immune infiltration in the keloid microenvironment. Through immunohistochemical verification of keloid and normal skin tissues, we identified immune-related features. Finally, four genes related to keloid (LGR5, PTN, JAG1, and DKK1) were identified, and we further studied keloid pathogenesis, which may provide new ideas and insights for keloid treatment. The design and workflow of this study are shown in **Figure 1**.

## Materials and methods

### Data acquisition

Three public datasets, GSE7890 (32), GSE92566 (33), and GSE44270 (34), from the Gene Expression Omnibus database (35) were retrospectively analyzed to obtain the corresponding data, as described previously (36). GSE7890 and GSE92566 were regarded as the training cohort, and GSE44270 was considered as the validation cohort (**Table 1**). Moreover, hallmark gene sets (37), which are coherently expressed signatures derived by aggregating many gene sets to represent well-defined biological states or processes, and immunologic signature gene sets (38), which represent cell states and perturbations within the immune system, were downloaded from the Molecular Signatures Database (version 7.4) (37, 39). Immune-related genes were obtained from ImmPort (40) (**Supplementary Table S1**).

**Table 1:** Details of cohorts.

	Training cohort		Validation cohort
	GSE7890	GSE92566	GSE44270
Last update date	March 25, 2019	March 25, 2019	July 26, 2018
Title	Gene profiling of keloid fibroblasts shows altered expression in multiple fibrosis-associated pathways	Aberrant connective tissue differentiation towards cartilage and bone underlies human keloid in African Americans	Keratinocyte and fibroblast gene expression in skin and keloid scar tissue
Design	5 keloid and 5 normal	4 keloid and 3 normal	18 keloid and 14 normal
GPL	GPL570 [HG-U133_Plus_2] Affymetrix Human Genome U133 Plus 2.0 Array	GPL570 [HG-U133_Plus_2] Affymetrix Human Genome U133 Plus 2.0 Array	GPL6244 [HuGene-1_0-st] Affymetrix Human Gene 1.0 ST Array [transcript (gene) version]

### Identification of differentially expressed genes (DEGs)

We preprocessed the original data of the training and validation cohorts using the *affy* package (version 1.68.0) (41) in R (version 3.6.2) to generate the corresponding gene expression profiles. DEGs between the keloid and normal groups were obtained using the *limma* package (version 3.46.0) (42) with a threshold of log_2_|fold change| > 1 and *P <*0.05. The *pheatmap* package (version 1.0.12) and the *ggplot2* package (version 3.3.3) were used for visualization.

### Identification of DEIGs

The DEGs obtained from the training cohort were crossed with immune-related genes to obtain the DEIGs. In addition, we computed the semantic similarity among Gene Ontology patterns to evaluate the functional enrichment similarity among DEIGs using the *GOSemSim* package (version 2.16.1) (43). At the same time, the STRING database (version 11.0) (44) was used to construct the protein–protein interaction (PPI) network. Moreover, we used the MCODE plug-in (version 1.32) (45) in Cytoscape (version 3.8.2) (46) to identify densely connected networks, as described previously (47, 48).

### Enrichment analysis

Each training cohort was divided into high and low groups according to the median expression of DEIGs, and the *limma* package was used for differential analysis. The *clusterProfiler* package (version 3.18.1) (49) was used for gene set enrichment analysis (50), disease ontology (51), gene ontology (52), and Kyoto Encyclopedia of Genes and Genomes (53) enrichment analysis to understand the biological processes involved in these DEIGs.

### Expression and distribution

We analyzed the expression and distribution of each DEIG based on the Human Protein Atlas (54) and Gene Expression Profiling Interactive Analysis (55) to gain a deeper understanding of each DEIG.

### Interactive network analysis

Furthermore, the interaction networks of DEIGs was analyzed from three aspects: DEIG–transcription factor, DEIG–microRNA (miRNA), and DEIG–drug through CHIP-X Enrichment Analysis Version 3 (56), Encyclopedia of RNA Interactomes (57), and Drug Gene Interaction Database (58), respectively. The interaction networks were visualized by Cytoscape (46).

### Immune infiltration analysis

We conducted immune infiltration landscape analysis based on CIBERSORT (59) to estimate the abundance of member cell types in a mixed keloid cell population using DEIG expression data in the training and validation cohorts.

### Clinical validation

Fifteen patients with keloid were enrolled for clinical validation. Keloid and adjacent normal skin tissue samples were collected. The study was approved by the ethics committee of Foshan First People’s Hospital (Foshan, China), and the patients provided signed written informed consent before enrollment. Immunohistochemistry assay was conducted according to the standard procedure (60, 61). The antibodies used were as follows: anti-JAG1 (1:150, SAB, United States), anti-DKK1 (1:150, SAB, United States), anti-PTN (1:150, SAB, United States), anti-LGR5 (1:1050, Affinity, United States), and secondary antibody conjugated with horseradish peroxidase (1:500, Affinity, United States). The process of histological scoring and analysis was conducted as described in our previous study (62).

## Results

### Identification of DEIGs

**Figure 2:**
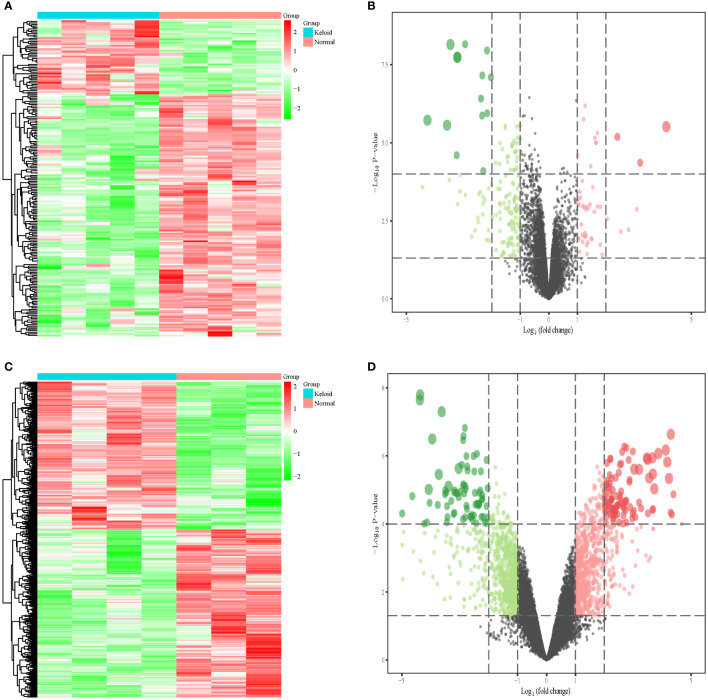
Differential expression analysis in three datasets. **(A)** Heat map of DEGs in GSE7890. **(B)** Volcano plot of DEGs in GSE7890. **(C)** Heat map of DEGs in GSE92566. **(D)** Volcano plot of DEGs in GSE92566.

**Figure 3:**
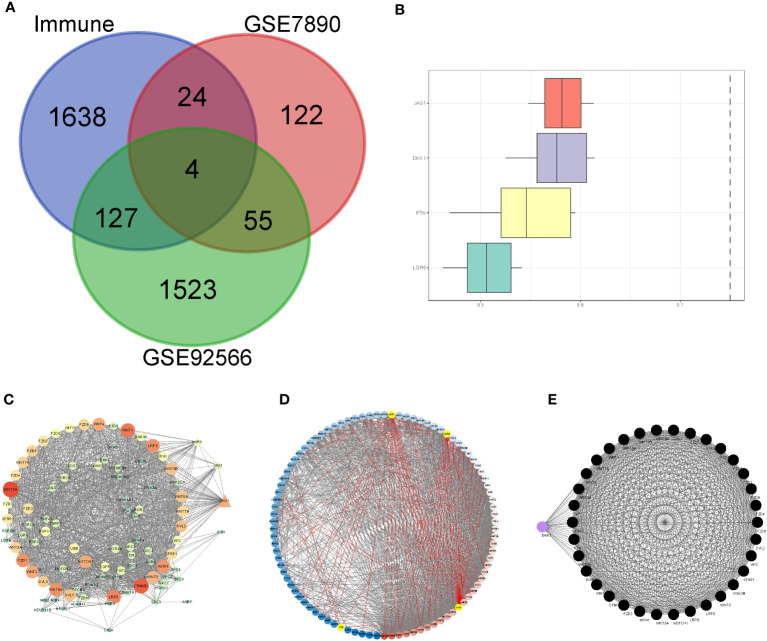
Principal component analysis and identification of differentially expressed immune-related gene (DEIGs). **(A)** Intersection of differentially expressed genes and immune-related genes. **(B)** Summary of the functional similarities of four DEIGs. **(C)** protein–protein interaction (PPI) network sized by degree. **(D)** PPI network colored by degree. **(E)** MCODE.

We included nine keloid samples and 8 normal samples in the training cohort and 18 keloid samples and 14 normal samples in the validation cohort, respectively (**Table 1**). There were 48 upregulated and 157 downregulated DEGs in GSE7890 (**Figures 2A**, **B**) as well as 801 upregulated DEGs and 908 downregulated DEGs in GSE92566 (**Figures 2C**, **D**). A total of 1,793 immune-related genes were obtained from ImmPort, and four DEIGs (LGR5, PTN, JAG1, and DKK1) were obtained by crossing the DEGs from the training cohort (**Figure 3A**). Detailed information on the four DEIGs is presented in **Table 2**. Despite the functional enrichment similarity, we found that the score of each DEIG was more than 0.5, and JAG1 had the highest score (**Figure 3B**). The PPI network with 94 nodes and 1,402 edges indicated the interaction of these genes (**Figure 3C**), and DKK1 may be the most important gene among these genes (**Figure 3D**). Moreover, using the MCODE plug-in, we observed DKK1 in a densely connected network with 35 nodes and 570 edges (**Figure 3E**).

**Table 2:** Details of DEIGs.

Gene symbol	Full name	Function	Pathway
LGR5	Leucine-rich repeat containing G protein-coupled receptor 5	Receptor for R-spondins that potentiates the canonical Wnt signaling pathway and acts as a stem cell marker of the intestinal epithelium and the hair follicle	GO:0004888 GO:0004930 GO:0005515 GO:0008528 GO:0016500
PTN	Pleiotrophin	Secreted growth factor that mediates its signal through cell surface proteoglycan and non-proteoglycan receptors	GO:0004864 GO:0005178 GO:0005515 GO:0005539 GO:0008083
JAG1	Jagged canonical notch ligand 1	Ligand for multiple Notch receptors and involved in the mediation of Notch signaling	GO:0005112 GO:0005198 GO:0005509 GO:0005515 GO:0005543
DKK1	Dickkopf WNT signaling pathway inhibitor 1	Antagonizes canonical Wnt signaling by inhibiting LRP5/6 interaction with Wnt and by forming a ternary complex with the transmembrane protein KREMEN that promotes internalization of LRP5/6	GO:0005515 GO:0008083 GO:0039706 GO:0048019 GO:0050750

### Enrichment analysis

**Figure 4:**
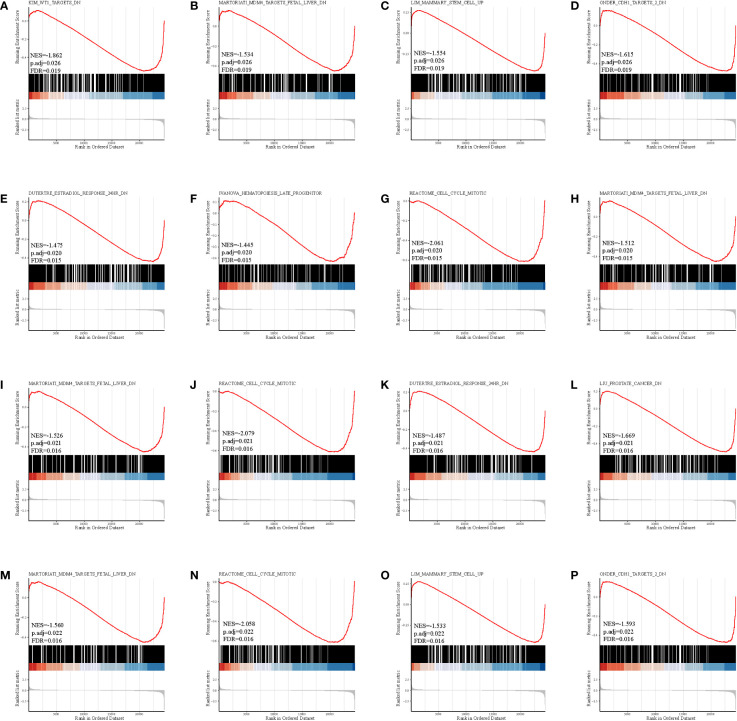
Gene set enrichment analysis in GSE7890. **(A–D)** DKK1, **(E–H)** JAG1, **(I–L)** LGR5, and **(M–P)** PTN.

**Figure 5:**
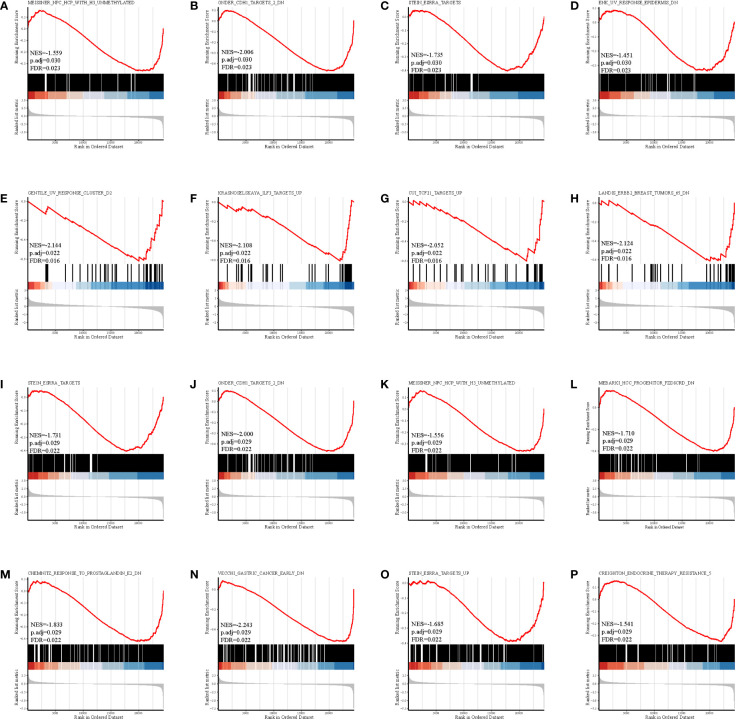
Gene set enrichment analysis in GSE92566. **(A–D)** DKK1, **(E–H)** JAG1, **(I–L)** LGR5, and **(M–P)** PTN.

**Figure 6:**
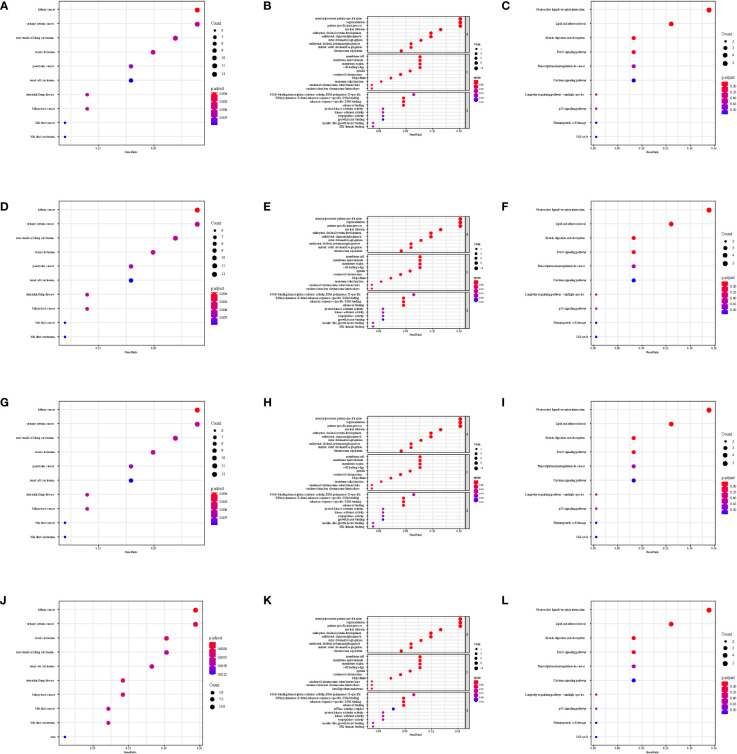
Disease Ontology, Gene Ontology, and Kyoto Encyclopedia of Genes and Genomes functional analysis in GSE7890. **(A–C)** DKK1, **(D–F)** JAG1, **(G–I)** LGR5, and **(J–L)** PTN.

**Figure 7:**
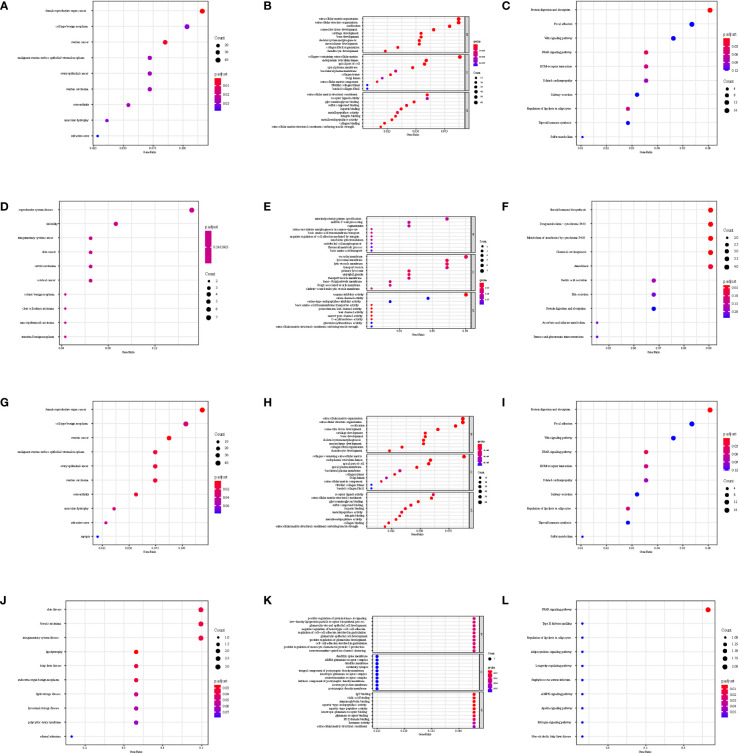
Disease Ontology, Gene Ontology, and Kyoto Encyclopedia of Genes and Genomes functional analysis in GSE92566. **(A–C)** DKK1, **(D–F)** JAG1, **(G–I)** LGR5, and **(J–L)** PTN.

The gene set enrichment analysis indicated that four DEIGs were mainly involved in stem cells and the cell cycle in GSE7890 (**Figure 4**) and UV response and therapy resistance in GSE92566 (**Figure 5**). Meanwhile, we conducted disease ontology, gene ontology, and Kyoto Encyclopedia of Genes and Genomes functional analyses in GSE7890 (**Figure 6**) and GSE92566 (**Figure 7**). The results indicated that DKK1, JAG1, LGR5, and PTN were enriched in immune-related terms (**Figures 6**, **7**).

### Expression and distribution

**Figure 8:**
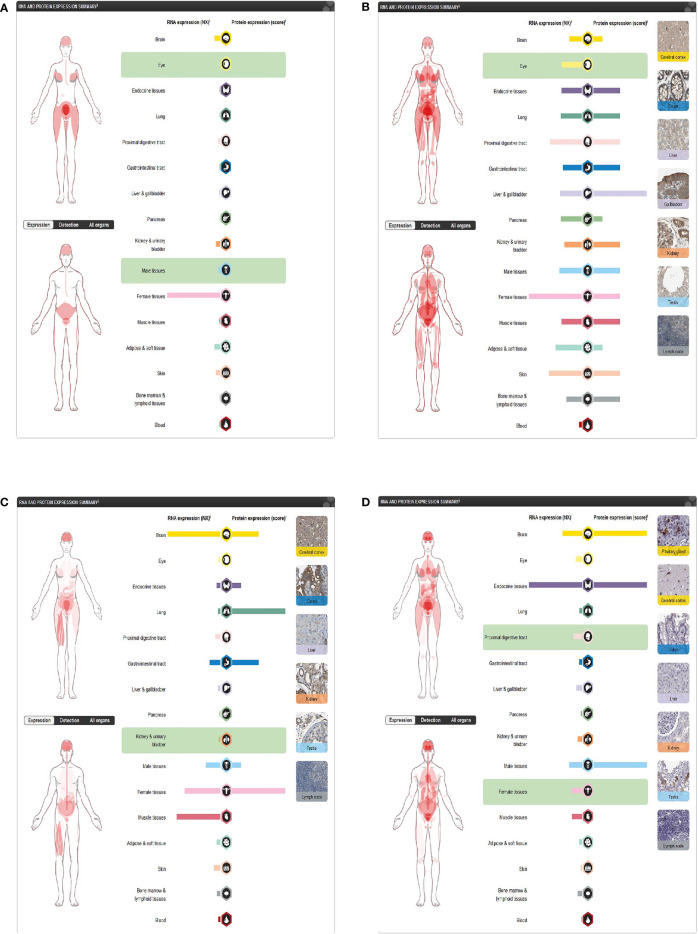
Expression of four differentially expressed immune-related genes. **(A)** DKK1 in the Human Protein Atlas. **(B)** JAG1 in the Human Protein Atlas. **(C)** LGR5 in the Human Protein Atlas. **(D)** PTN in the Human Protein Atlas.

Through the Human Protein Atlas, we found that LGR5, PTN, JAG1, and DKK1 were expressed in the skin (**Figures 8A**–**D**).

### Interactive network analysis

**Figure 9:**
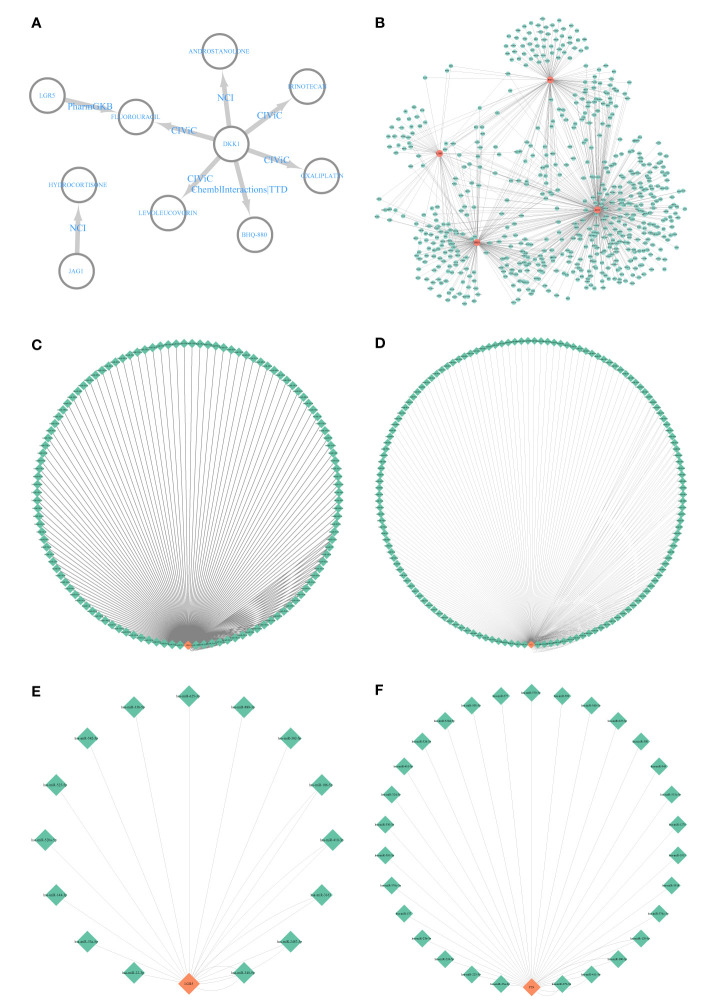
Interactive network analysis. **(A)** Differentially expressed immune-related gene (DEIG)–drug network; **(B)** DEIG–transcription factor network. **(C)** DKK1–miRNA network. **(D)** JAG1–miRNA network. **(E)** LGR5–miRNA network. **(F)** PTN–miRNA network.

The DEIG–drug network showed that JAG1 was involved in hydrocortisone, and DKK1 and LGR5 were involved in fluorouracil (**Figure 9A**). Besides this, DKK1 was also involved in androstanolone, irinotecan, oxaliplatin, BHQ-880 (anti-DKK1 mAb), and lecoleucovorin (**Figure 9A**). The DEIG–transcription factor network indicated that the four DEIGs had the same transcription factors (**Figure 9B**). The DEIG–miRNA networks also demonstrated the same miRNAs (**Figures 9C–F**).

### Immune infiltration analysis

**Figure 10:**
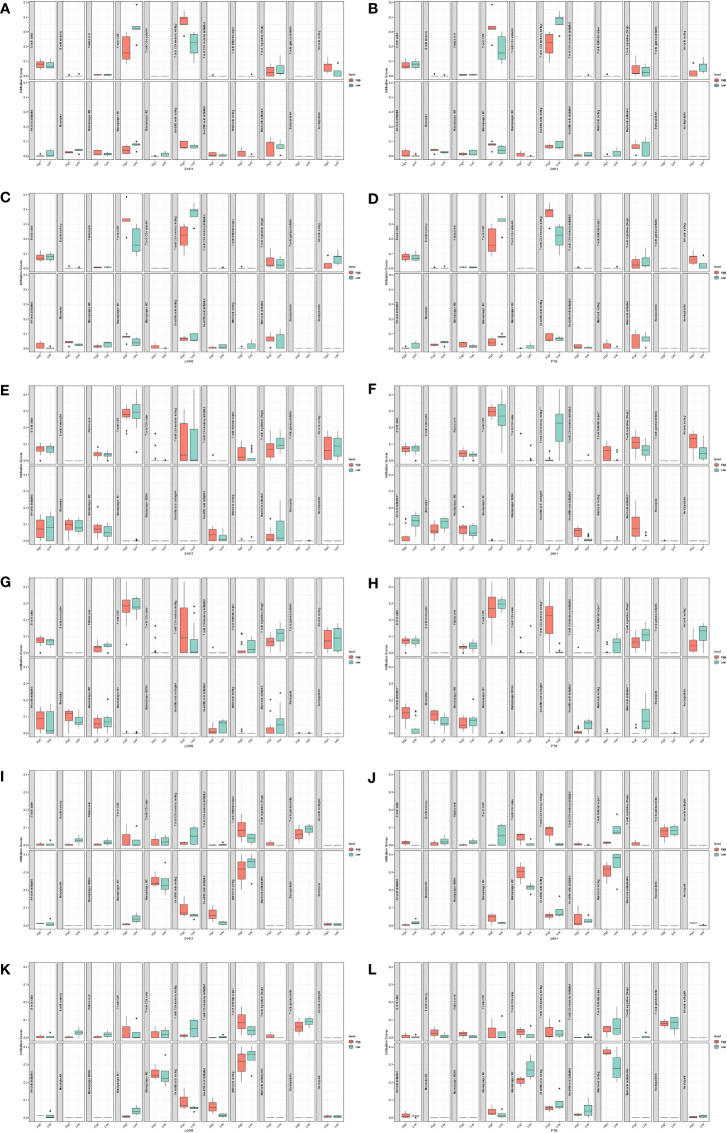
Immune infiltration analysis. **(A)** DKK1 in GSE7890. **(B)** JAG1 in GSE7890. **(C)** LGR5 in GSE7890. **(D)** PTN in GSE7890. **(E)** DKK1 in GSE44270. **(F)** JAG1 in GSE44270. **(G)** LGR5 in GSE44270. **(H)** PTN in GSE44270. **(I)** DKK1 in GSE92566. **(J)** JAG1 in GSE92566. **(K)** LGR5 in GSE92566. **(L)** PTN in GSE92566.

Using CIBERSORT, we found that CD8^+^ T cells infiltrated the low-DKK1 group, resting memory CD4^+^ T cells infiltrated the high-DKK1 group, and M1 macrophages infiltrated the low-DKK1 group in GSE7890 (**Figure 10A**). CD8^+^ T cells infiltrated the high-JAG1 group, resting memory CD4^+^ T cells infiltrated the low-JAG1 group, and M1 macrophages infiltrated the high-JAG1 group in GSE7890 (**Figure 10B**). CD8^+^ T cells infiltrated the high-LGR5 group, resting memory CD4^+^ T cells infiltrated the low-LGR5 group, and M1 macrophages infiltrated the high-LGR5 group in GSE7890 (**Figure 10C**). CD8^+^ T cells infiltrated the low-PTN group, resting memory CD4^+^ T cells infiltrated the high-PTN group, and M1 macrophages infiltrated the low-PTN group in GSE7890 (**Figure 10D**). Interestingly, similar results were obtained for GSE44270 and GSE92566 (**Figures 10E**–**L**).

### Preliminary immunohistochemistry validation

**Figure 11:**
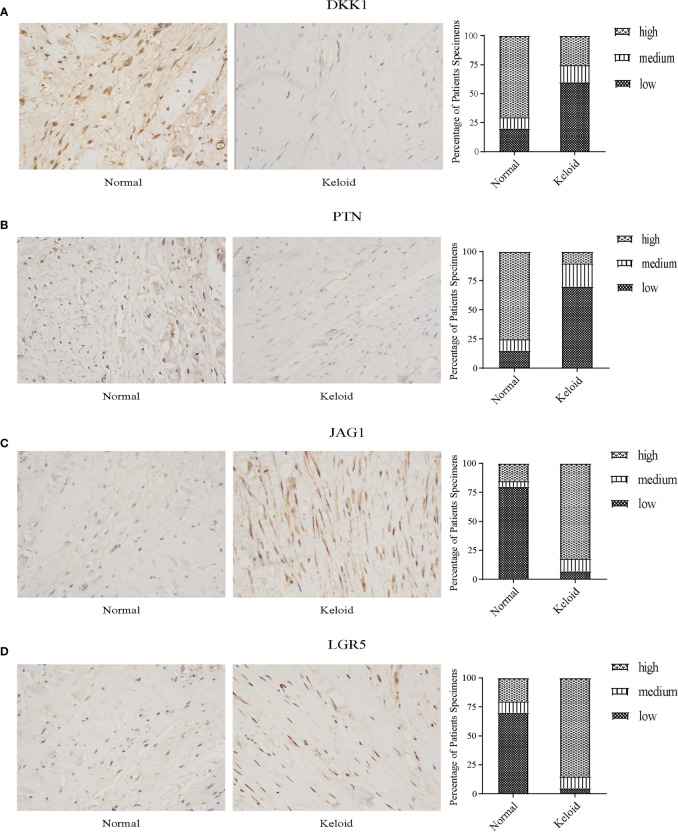
Immunohistochemistry staining and histologic scoring. **(A)** Expression of DKK1 in normal tissue and keloid. **(B)** Expression of PTN in normal tissue and keloid. **(C)** Expression of JAG1 in normal tissue and keloid. **(D)** Expression of LGR5 in normal tissue and keloid.

Through immunohistochemistry staining, we found that the expression of DKK1 and PTN was lower in the keloid (**Figures 11A**, **B**), while JAG1 and LGR5 showed a higher expression in the keloid (**Figures 11C**, **D**). In the keloid tissue, the brownish-yellow particles with a positive expression of the four molecules were mainly distributed in the nucleus and cytoplasm of fibroblasts. In the normal tissues, the brownish-yellow particles with positive expression of the four molecules were mainly distributed in the nucleus and cytoplasm of fibroblasts (**Figure 11**).

## Discussion

Keloid is formed from collagen that is produced during a protracted wound healing process (63). They can be divided into the exudation, collagen, and maturation stages (64). In the exudation stage, necrotic tissue is cleared through phagocytosis, removal, and absorption, and the wound is bonded together by the network structure formed by cellulose. In the collagen fiber stage, fibroblasts polymerize to form collagen fibers with the participation of glycine, hydroxyproline, and other substances, which increase the tensile strength of the wound. In the mature stage, collagen fibers are continuously synthesized. At the same time, they are constantly decomposed under the action of collagenase, resulting in degenerative changes in skin scars. Most previous studies have focused on fibroblasts and collagen, but few have emphasized the importance of immunity in keloid Despite intensive research, treatment remains empirical and unsatisfactory. Therefore, it is necessary to identify diagnostic markers that inhibit keloid proliferation and reduce keloid formation. We conducted a comprehensive bioinformatics analysis of 27 keloid samples and 22 normal tissue samples and finally determined that four immune-related signatures were closely related to keloid, and these signatures were related to immunity, stress, and inflammation. A previous study demonstrated that immune-related cells, such as T cells, Th2 cells, and Th1 cells, were upregulated in lesional *versus* normal skin (65). Here we aimed to explore the potential pathogenesis of keloid-associated immune molecules. We further explained the important role of immunity in keloid through multiple dataset analyses. We found that the expression of DKK1 and PTN was downregulated, while the expression of JAG1 and LGR5 was upregulated in keloid. We obtained the same conclusion through immunohistochemistry. This provides an important reference for future scientific research. We can promote or inhibit the expression of these four molecules through corresponding mechanisms to inhibit the growth of keloid, which will contribute to follow-up clinical research.

The immune system plays an important role in preventing pathogen infiltration, inducing inflammation, initiating downstream processes, and recruiting fibrocytes in keloid (66, 67). Keloid formation is usually considered to be the result of long-term proliferation and a delayed remodeling stage, which is caused by increased fibroblast proliferation and excessive collagen deposition (68, 69). In the dermal thickening of keloid, versican, syndecan-1, fibronectin, thrombospondin-1, tenascin C, CD44, integrin β1, and N-cadherin were immunolocalized in the elongated fibroblasts that were close to the immune cell infiltrate, attached to collagen bundles, and around the microvasculature as well as in some immune cells. Galectin-1/3 was present in the cytoplasm and along the cell membrane of some immune cells and fibroblasts, suggesting that galectin-1/3, in concert with some of the extracellular matrix molecules, was produced to counteract the immune response in keloid. Recent studies have found that the degree of CD3^+^ cell infiltration in keloid tissue was higher (70). The number of T cells in the tissue is significantly higher than that in peripheral blood and is related to the size of the keloid, suggesting that the occurrence of a keloid is closely related to the role of T cells (71, 72). Macrophages also play an important role in keloid formation. There are two types of macrophages: M1 and M2. M1 plays a role in the early stages of keloid formation, while M2 plays a role in the late stages of keloid formation. In the process of keloid formation, M1 constantly transforms into M2, resulting in the accumulation of a large number of collagen fibers; however, the specific mechanism needs to be further clarified (12). At the same time, we often use glucocorticoids, such as triamcinolone acetonide, locally to treat keloid. Triamcinolone acetonide inhibits the migration of inflammatory cells and reduces fibroblast activity. Most importantly, it can induce lymphocyte DNA degradation and induce lymphocyte apoptosis, thus playing an immunosuppressive role (71, 72). In conclusion, the immune system plays an indispensable role in keloid development. Additionally, keloid formation is closely related to inflammatory responses, especially inflammation-related factors, such as interleukin 6, transforming growth factor-β, and tumor necrosis factor-α and inflammation-related cells, such as neutrophils, mast cells, and lymphocytes (73). The inflammatory response is an adaptive response to adverse stimuli. It can promote fibroblast proliferation and collagen synthesis and eventually develop into a keloid. However, the relationship between inflammatory response and keloid is still in the scientific research stage, and research on the specific mechanism and transformation of the clinical application needs to be further explored.

Here we first focused on immune-related genes and obtained four DEIGs after merging them with the DEGs of GSE7890 and GSE92566, namely LGR5, PTN, JAG1, and DKK1. LGR5 encodes a component of the Wnt receptor complex, which specifically acts as a receptor for a family of Wnt signal agonists named R-spondins. LGR5 is a newly discovered Wnt signal transduction target gene. It is considered a tumor stem cell marker. It is often overexpressed in tumor cells and is strongly associated with tumor formation and development. However, the specific mechanism between LGR5 and the immune-related tumor microenvironment is unclear (74–76). Through the study of keloid in the immunosuppressive microenvironment, exogenous TGF-β1 was induced and LGR5 was highly expressed, but the specific mechanism was not further confirmed. However, this is a potential emerging research direction, which provides new insights for targeted immunotherapy in the future (77). PTN is a member of the heparin-binding cytokine family, which is highly conserved, secretes proteins related to the extracellular matrix, and plays a significant role in cell growth and survival, cell migration, angiogenesis, wound healing, and tumorigenesis (78–80). PTN combined with midkine inactivates the phosphatase activity of receptor protein tyrosine phosphatase to trigger immune responses (81). Huang *et al.* found that PTN is one of the seven significantly dysregulated genes, which is downregulated in human keloid, but most other genes, such as TGF-β1, TGF-β3, and various collagens, are upregulated in keloid (82, 83). Lee et al. further found that, in keloid, not only the expression of PTN decreased but also the expression of its functional receptor RPTPB/F and upstream regulators was decreased, including PDGF-B, PDGFR-A, and PDGFR-B, which may be a potential novel treatment for keloid in humans (32, 84). JAG1, a ligand for the Notch1 receptor, is involved in Notch signaling. JAG1 participates in the regulation of homeostasis in human tissues and plays an important role in the proliferation, migration, and invasion of fibroblasts (85–87), which can lead to changes in Notch receptor expression in keloid (88). It was found that JAG1 was significantly overexpressed at the mRNA and protein levels in keloid as well as in immune cells. More than 30 years ago, Russell et al. used quantitative real-time polymerase chain reaction to detect the expression level of keloid tissue and normal skin tissue at the same site; increased JAG1 expression was observed in the fibroblasts of keloid nodules (89). Resistance to related drugs, such as hydrocortisone, can be observed in fibroblasts of keloid nodules, which can change the chemistry and morphology of connective tissue cells and hinder the production of intercellular substances, such as collagen fibers. Hydrocortisone can be used to treat keloid by targeting JAG1 (90, 91). DKK1 is an inhibitor of the Wnt signaling pathway, which can regulate Wnt through negative feedback and participates in tumor cell proliferation, apoptosis, migration, and angiogenesis (92, 93). DKK1 binds to the LRP6 co-receptor and inhibits β-catenin-dependent Wnt signaling, and the downregulation of β-catenin is crucial to human immune function (94, 95). Furthermore, DKK1 can regulate immune cells and suppress immune reactions through the GSK3β/E2F1/T-bet axis in CD8^+^ T cells (96, 97). In a series of studies, it was found that the expression of DKK1 decreased in tumor cells. Keloid is benign skin tumor; therefore, it can be inferred that DKK1 is present at a low level in keloid, which was also confirmed by our previous data analysis and experimental detection (98–100). Moreover, DKK1 regulates the activity of immune cells and participates in T-cell differentiation. Therefore, DKK1 is a promising target for keloid immunotherapy. DKK1 has attracted extensive attention as a mechanism for keloid and immune cells (101). Moreover, in immune infiltration, we identified some immune cells, which were consistent with the available source (67, 102, 103). In a word, we established that four DEIGs played a vital role in the pathogenesis and treatment of keloid; however, the specific mechanism of these four genes in keloid has not been studied. Therefore, it is necessary to conduct further analyses to evaluate the effects of these genes.

The current study has several highlights. First, few studies have linked keloid to immune-related molecules. The expression profiles of normal skin and keloid tissues help us gain a comprehensive understanding of their pathological processes and identify keloid diagnostic biomarkers. Second, DEGs are used to screen immune molecules, which have special advantages in gene expression datasets because they can estimate the connectivity between modules and clinical features. However, our study had some limitations. First, even though we included three datasets, the sample size was small. The data used in this study was obtained from different platforms. Despite the fact that homogenization was conducted, the source bias still be ignored. Finally, this was an *in silico* analysis, and there is an urgent need for molecular biology experiments and prospective, well-designed, multicenter studies to verify these findings. The specific role of these biomarkers in keloid, their specific association with clinical features (keloid in different periods), and their diagnostic role require further investigation and verification in a large number of clinical patients.

## Data availability statement

The datasets presented in this study can be found in online repositories. The names of the repositories and accession numbers can be found in the article.

## Ethics statement

Ethical approval was obtained from the review committee of Foshan First People’s Hospital, written informed consent was obtained from the participants prior to sample collection.

## Author contributions

XC, SZ, RY, XW, and BL conceived, designed, or planned the idea. XW, BL, JL, XP, PZ, and XZ collected the data. XC, SZ, RY, XW, and BL analyzed the data. All authors interpreted the results. XW and BL drafted the manuscript, and XC, SZ, and RY revised the manuscript. All authors contributed to the article and approved the submitted version.

## Funding

This study was supported by the National Natural Science Foundation of China (82002913), Guangdong Basic and Applied Basic Research Foundation (2021A1515011453, 2022A1515012160, 2021B1515120036, and 2022A1515012245) and the Medical Scientific Research Foundation of Guangdong Province (A2022293).

## Acknowledgments

We thank the databases mentioned in our study.

## Conflict of interest

The authors declare that the research was conducted in the absence of any commercial or financial relationships that could be construed as a potential conflict of interest.

## Publisher’s note

All claims expressed in this article are solely those of the authors and do not necessarily represent those of their affiliated organizations, or those of the publisher, the editors and the reviewers. Any product that may be evaluated in this article, or claim that may be made by its manufacturer, is not guaranteed or endorsed by the publisher.

## Supplementary material

Supplementary Table 1 Details of the immune-related genes.

## Abbreviations

DEG, Differentially expressed gene; DEIG, Differentially expressed immune-related gene; DKK1, Dickkopf WNT signaling pathway inhibitor 1; JAG1, Jagged canonical notch ligand 1; LGR5, Leucine-rich repeat containing G protein-coupled receptor 5; miRNA, microRNA; PPI, Protein–protein interaction; PTN, Pleiotrophin.

## References

[1] Appleton I Brown N Willoughby DA. Apoptosis, necrosis, and proliferation: possible implications in the etiology of keloids. Am J Pathol (1996) 149(5):1441–7. PMC1865267 8909233

[2] Huang C Akaishi S Hyakusoku H Ogawa R. Are keloid and hypertrophic scar different forms of the same disorder? a fibroproliferative skin disorder hypothesis based on keloid findings. Int Wound J (2014) 11(5):517–22. doi: 10.1111/j.1742-481X.2012.01118.x PMC7950391 23173565

[3] Niessen FB Spauwen PH Schalkwijk J Kon M. On the nature of hypertrophic scars and keloids: a review. Plast Reconstr Surg (1999) 104(5):1435–58. doi: 10.1097/00006534-199910000-00031 10513931

[4] Fong CY Biswas A Subramanian A Srinivasan A Choolani M Bongso A. Human keloid cell characterization and inhibition of growth with human wharton's jelly stem cell extracts. J Cell Biochem (2014) 115(5):826–38. doi: 10.1002/jcb.24724 24265231

[5] Rabello FB Souza CD Farina Junior JA. Update on hypertrophic scar treatment. Clinics (Sao Paulo) (2014) 69(8):565–73. doi: 10.6061/clinics/2014(08)11 PMC4129552 25141117

[6] Ogawa R Dohi T Tosa M Aoki M Akaishi S. The latest strategy for keloid and hypertrophic scar prevention and treatment: The Nippon medical school (NMS) protocol. J Nippon Med School (2021) 88(1):2–9. doi: 10.1272/jnms.JNMS.2021_88-106 32741903

[7] Zhang T Wang X-F Wang Z-C Lou D Fang Q-Q Hu Y-Y. Current potential therapeutic strategies targeting the TGF-β/Smad signaling pathway to attenuate keloid and hypertrophic scar formation. Biomed Pharmacother (2020) 129, 110287. doi: 10.1016/j.biopha.2020.110287 32540643

[8] Yagmur C Akaishi S Ogawa R Guneren E. Mechanical receptor–related mechanisms in scar management: A review and hypothesis. Plast Reconstr Surg (2010) 126(2):426–34. doi: 10.1097/PRS.0b013e3181df715d 20375759

[9] Wang X-Q Song F Liu Y-K. Hypertrophic scar regression is linked to the occurrence of endothelial dysfunction. PloS One (2017) 12(5):e0176681. doi: 10.1371/journal.pone.0176681 28472181 PMC5417599

[10] Bond JS Duncan JAL Mason T Sattar A Boanas A O’Kane S. Scar redness in humans: How long does it persist after incisional and excisional wounding? Plast Reconstructive Surg (2008) 121(2):487–96. doi: 10.1097/01.prs.0000299183.88334.37 18300967

[11] Wang Z-C Zhao W-Y Cao Y Liu Y-Q Sun Q Shi P. The roles of inflammation in keloid and hypertrophic scars. Front Immunol (2020), 11, 603187. doi: 10.3389/fimmu.2020.603187 33343575 PMC7746641

[12] Williams EA Thaller SR. The role of fat grafting in the treatment of keloid scars and venous ulcers. J Craniofac Surg (2019) 30(3):696–7. doi: 10.1097/SCS.0000000000005208 31048608

[13] Scala J Vojvodic A Vojvodic P Vlaskovic-Jovicevic T Peric-Hajzler Z Matovic D. Botulin toxin use in Scars/Keloids treatment. Open Access Maced J Med Sci (2019) 7(18):2979–81. doi: 10.3889/oamjms.2019.783 PMC6910812 31850103

[14] Brown BC McKenna SP Siddhi K McGrouther DA Bayat A. The hidden cost of skin scars: quality of life after skin scarring. J Plast Reconstr Aesthet Surg (2008) 61(9):1049–58. doi: 10.1016/j.bjps.2008.03.020 18617450

[15] Bayat A McGrouther DA Ferguson MW. Skin scarring. BMJ (2003) 326(7380):88–92. doi: 10.1136/bmj.326.7380.88 12521975 PMC1125033

[16] Berman B Maderal A Raphael B. Keloids and hypertrophic scars: Pathophysiology, classification, and treatment. Dermatol Surg (2017), 43, S3–S18. doi: 10.1097/DSS.0000000000000819 27347634

[17] Lee JY-Y Yang C-C Chao S-C Wong T-W. Histopathological differential diagnosis of keloid and hypertrophic scar. Am J Dermatopathol (2004) 26(5):379–84. doi: 10.1097/00000372-200410000-00006 15365369

[18] Gold MH Berman B Clementoni MT Gauglitz GG Nahai F Murcia C. Updated international clinical recommendations on scar management: part 1–evaluating the evidence. Dermatol Surg (2014) 40(8):817–24. doi: 10.1111/dsu.0000000000000049 25068543

[19] Gold MH McGuire M Mustoe TA Pusic A Sachdev M Waibel J. Updated international clinical recommendations on scar management: part 2–algorithms for scar prevention and treatment. Dermatol Surg (2014) 40(8):825–31. doi: 10.1111/dsu.0000000000000050 25068544

[20] Kim S Choi TH Liu W Ogawa R Suh JS Mustoe TA. Update on scar management: guidelines for treating Asian patients. Plast Reconstr Surg (2013) 132(6):1580–9. doi: 10.1097/PRS.0b013e3182a8070c 24281584

[21] Janssen de Limpens AM Cormane RH. Studies on the immunologic aspects of keloids and hypertrophic scars. Arch Dermatol Res (1982) 274(3-4):259–66. doi: 10.1007/BF00403728 6187300

[22] Kischer CW Shetlar MR Shetlar CL Chvapil M. Immunoglobulins in hypertrophic scars and keloids. Plast Reconstr Surg (1983) 71(6):821–5. doi: 10.1097/00006534-198306000-00015 6344114

[23] Bagabir R Byers RJ Chaudhry IH Muller W Paus R Bayat A. Site-specific immunophenotyping of keloid disease demonstrates immune upregulation and the presence of lymphoid aggregates. Br J Dermatol (2012) 167(5):1053–66. doi: 10.1111/j.1365-2133.2012.11190.x 23106354

[24] Jiao H Fan J Cai J Pan B Yan L Dong P. Analysis of characteristics similar to autoimmune disease in keloid patients. Aesthetic Plast Surg (2015) 39(5):818–25. doi: 10.1007/s00266-015-0542-4 26296635

[25] Klotz T Munn Z Aromataris EC Greenwood JE. Imiquimod to prevent keloid recurrence postexcision: A systematic review and meta-analysis. Wound Repair Regener (2020) 28(1):145–56. doi: 10.1111/wrr.12766 31587416

[26] Sandulache VC Parekh A Li-Korotky H Dohar JE Hebda PA. Prostaglandin E2 inhibition of keloid fibroblast migration, contraction, and transforming growth factor (TGF)-beta1-induced collagen synthesis. Wound Repair Regener (2007) 15(1):122–33. doi: 10.1111/j.1524-475X.2006.00193.x 17244328

[27] Zhang L Luo H Meng W Cen Y Huang Q Li H. Integration of flow cytometry and computational analysis to dissect the epidermal cellular subsets in keloids that correlate with recurrence. J Invest Dermatol (2021) 141, 2521–29e4. doi: 10.1016/j.jid.2021.03.022 33839145

[28] Shen R Li P Li B Zhang B Feng L Cheng S. Identification of distinct immune subtypes in colorectal cancer based on the stromal compartment. Front Oncol (2019) 91497:1497. doi: 10.3389/fonc.2019.01497 PMC6965328 31998649

[29] Tang L Peng C Zhu SS Zhou Z Liu H Cheng Q. Tre2-Bub2-Cdc16 family proteins based nomogram serve as a promising prognosis predicting model for melanoma. Front Oncol (2020) 10579625:579625. doi: 10.3389/fonc.2020.579625 PMC7656061 33194704

[30] Zhang Z Li J He T Ding J. Bioinformatics identified 17 immune genes as prognostic biomarkers for breast cancer: Application study based on artificial intelligence algorithms. Front Oncol (2020) 10330:330. doi: 10.3389/fonc.2020.00330 PMC7137378 32296631

[31] Zhao J Guo C Xiong F Yu J Ge J Wang H. Single cell RNA-seq reveals the landscape of tumor and infiltrating immune cells in nasopharyngeal carcinoma. Cancer Lett (2020), 10, 330. doi: 10.1016/j.canlet.2020.02.010 32061950

[32] Smith JC Boone BE Opalenik SR Williams SM Russell SB. Gene profiling of keloid fibroblasts shows altered expression in multiple fibrosis-associated pathways. J Invest Dermatol (2008) 128(5):1298–310. doi: 10.1038/sj.jid.5701149 PMC2933038 17989729

[33] Fuentes-Duculan J Bonifacio KM Suarez-Farinas M Kunjravia N Garcet S Cruz T. Aberrant connective tissue differentiation towards cartilage and bone underlies human keloids in African americans. Exp Dermatol (2017) 26(8):721–7. doi: 10.1111/exd.13271 PMC5466890 27943413

[34] Hahn JM Glaser K McFarland KL Aronow BJ Boyce ST Supp DM. Keloid-derived keratinocytes exhibit an abnormal gene expression profile consistent with a distinct causal role in keloid pathology. Wound Repair Regener (2013) 21(4):530–44. doi: 10.1111/wrr.12060 23815228

[35] Barrett T Wilhite SE Ledoux P Evangelista C Kim IF Tomashevsky M. NCBI GEO: archive for functional genomics data sets–update. Nucleic Acids Res (2013) 41(Database issue):D991–995. doi: 10.1093/nar/gks1193 PMC3531084 23193258

[36] Zhou JG Liang B Jin SH Liao HL Du GB Cheng L. Development and validation of an RNA-Seq-Based prognostic signature in neuroblastoma. Front Oncol (2019) 91361:1361. doi: 10.3389/fonc.2019.01361 PMC6904333 31867276

[37] Liberzon A Birger C Thorvaldsdottir H Ghandi M Mesirov JP Tamayo P. The molecular signatures database (MSigDB) hallmark gene set collection. Cell Syst (2015) 1(6):417–25. doi: 10.1016/j.cels.2015.12.004 PMC4707969 26771021

[38] Godec J Tan Y Liberzon A Tamayo P Bhattacharya S Butte AJ. Compendium of immune signatures identifies conserved and species-specific biology in response to inflammation. Immunity (2016) 44(1):194–206. doi: 10.1016/j.immuni.2015.12.006 26795250 PMC5330663

[39] Subramanian A Tamayo P Mootha VK Mukherjee S Ebert BL Gillette MA. Gene set enrichment analysis: a knowledge-based approach for interpreting genome-wide expression profiles. Proc Natl Acad Sci U.S.A. (2005) 102(43):15545–50. doi: 10.1073/pnas.0506580102 PMC1239896 16199517

[40] Bhattacharya S Dunn P Thomas CG Smith B Schaefer H Chen J. ImmPort, toward repurposing of open access immunological assay data for translational and clinical research. Sci Data (2018) 5, 80015. doi: 10.1038/sdata.2018.15 PMC5827693 29485622

[41] Gautier L Cope L Bolstad BM Irizarry RA. Affy–analysis of affymetrix GeneChip data at the probe level. Bioinformatics (2004) 20(3):307–15. doi: 10.1093/bioinformatics/btg405 14960456

[42] Ritchie ME Phipson B Wu D Hu Y Law CW Shi W. Limma powers differential expression analyses for RNA-sequencing and microarray studies. Nucleic Acids Res (2015) 43(7):e47. doi: 10.1093/nar/gkv007 25605792 PMC4402510

[43] Yu G Li F Qin Y Bo X Wu Y Wang S. GOSemSim: an r package for measuring semantic similarity among GO terms and gene products. Bioinformatics (2010) 26(7):976–8. doi: 10.1093/bioinformatics/btq064 20179076

[44] von Mering C Huynen M Jaeggi D Schmidt S Bork P Snel B. STRING: a database of predicted functional associations between proteins. Nucleic Acids Res (2003) 31(1):258–61. doi: 10.1093/nar/gkg034 PMC165481 12519996

[45] Bader GD andHogue CWV. An automated method for finding molecular complexes in large protein interaction networks. BMC Bioinf (2003) 4, 2. doi: 10.1186/1471-2105-4-2 PMC149346 12525261

[46] Shannon P Markiel A Ozier O Baliga NS Wang JT Ramage D. Cytoscape: a software environment for integrated models of biomolecular interaction networks. Genome Res (2003) 13(11):2498–504. doi: 10.1101/gr.1239303 PMC403769 14597658

[47] Liang B Zhang XX Gu N. Virtual screening and network pharmacology-based synergistic mechanism identification of multiple components contained in guanxin V against coronary artery disease. BMC Complement Med Ther (2020) 20(1):345. doi: 10.1186/s12906-020-03133-w 33187508 PMC7664106

[48] Liang B Liang Y Gu N. Pharmacological mechanisms of sodium-glucose co-transporter 2 inhibitors in heart failure with preserved ejection fraction. BMC Cardiovasc Disord (2022) 22(1):261. doi: 10.1186/s12872-022-02693-8 35689186 PMC9188076

[49] Yu G Wang LG Han Y He QY. clusterProfiler: an r package for comparing biological themes among gene clusters. OMICS (2012) 16(5):284–7. doi: 10.1089/omi.2011.0118 PMC3339379 22455463

[50] Hanzelmann S Castelo R Guinney J. GSVA: gene set variation analysis for microarray and RNA-seq data. BMC Bioinf (2013) 14, 7. doi: 10.1186/1471-2105-14-7 PMC3618321 23323831

[51] Bello SM Shimoyama M Mitraka E Laulederkind SJF Smith CL Eppig JT. Disease ontology: improving and unifying disease annotations across species. Dis Model Mech (2018) 11(3). doi: 10.1242/dmm.032839 PMC5897730 29590633

[52] Ashburner M Ball CA Blake JA Botstein D Butler H Cherry JM. Gene ontology: tool for the unification of biology. Gene Ontol Consortium Nat Genet (2000) 25(1):25–9. doi: 10.1038/75556 PMC3037419 10802651

[53] Kanehisa M Furumichi M Tanabe M Sato Y Morishima K. KEGG: new perspectives on genomes, pathways, diseases and drugs. Nucleic Acids Res (2017) 45(D1):D353–61. doi: 10.1093/nar/gkw1092 PMC5210567 27899662

[54] Uhlen M Fagerberg L Hallstrom BM Lindskog C Oksvold P Mardinoglu A. Proteomics. tissue-based map of the human proteome. Science (2015) 347(6220):1260419. doi: 10.1126/science.1260419 25613900

[55] Tang Z Li C Kang B Gao G Li C Zhang Z. GEPIA: a web server for cancer and normal gene expression profiling and interactive analyses. Nucleic Acids Res (2017) 45(W1):W98–W102. doi: 10.1093/nar/gkx247 28407145 PMC5570223

[56] Keenan AB Torre D Lachmann A Leong AK Wojciechowicz ML Utti V. ChEA3: transcription factor enrichment analysis by orthogonal omics integration. Nucleic Acids Res (2019) 47(W1):W212–24. doi: 10.1093/nar/gkz446 PMC6602523 31114921

[57] Li JH Liu S Zhou H Qu LH Yang JH. starBase v2.0: decoding miRNA-ceRNA, miRNA-ncRNA and protein-RNA interaction networks from large-scale CLIP-seq data. Nucleic Acids Res (2014) 42(Database issue):D92–97. doi: 10.1093/nar/gkt1248 PMC3964941 24297251

[58] Cotto KC Wagner AH Feng YY Kiwala S Coffman AC Spies G. DGIdb 3.0: a redesign and expansion of the drug-gene interaction database. Nucleic Acids Res (2018) 46(D1):D1068–73. doi: 10.1093/nar/gkx1143 PMC5888642 29156001

[59] Chen B Khodadoust MS Liu CL Newman AM Alizadeh AA. Profiling tumor infiltrating immune cells with CIBERSORT. Methods Mol Biol (2018), 1711, 243–59. doi: 10.1007/978-1-4939-7493-1_12 PMC5895181 29344893

[60] Liang B Zhang X-X Li R Gu N. Guanxin V protects against ventricular remodeling after acute myocardial infarction through the interaction of TGF-β1 and vimentin. Phytomedicine (2022), 95, 153866. doi: 10.1016/j.phymed.2021.153866 34883417

[61] Zhang X-X Shao C-L Cheng S-Y Zhu Y Liang B Gu N. Effect of guanxin V in animal model of acute myocardial infarction. BMC Complement Med Ther (2021) 21(1):72. doi: 10.1186/s12906-021-03211-7 33618704 PMC7898759

[62] Zhou S Liang Y Zhang X Liao L Yang Y Ouyang W. SHARPIN promotes melanoma progression *via* Rap1 signaling pathway. J Invest Dermatol (2020) 140(2):395–403 e396. doi: 10.1016/j.jid.2019.07.696 31401046

[63] Limandjaja GC Niessen FB Scheper RJ Gibbs S. The keloid disorder: Heterogeneity, histopathology, mechanisms and models. Front Cell Dev Biol (2020) 8360:360. doi: 10.3389/fcell.2020.00360 PMC7264387 32528951

[64] Monstrey S Middelkoop E Vranckx JJ Bassetto F Ziegler UE Meaume S. Updated scar management practical guidelines: non-invasive and invasive measures. J Plast Reconstr Aesthet Surg (2014) 67(8):1017–25. doi: 10.1016/j.bjps.2014.04.011 24888226

[65] Wu J Del Duca E Espino M Gontzes A Cueto I Zhang N. RNA Sequencing keloid transcriptome associates keloids with Th2, Th1, Th17/Th22, and JAK3-skewing. Front Immunol (2020) 11597741:597741. doi: 10.3389/fimmu.2020.597741 PMC7719808 33329590

[66] Chen Y Jin Q Fu X Qiao J Niu F. Connection between T regulatory cell enrichment and collagen deposition in keloid. Exp Cell Res (2019) 383(2):111549. doi: 10.1016/j.yexcr.2019.111549 31400303

[67] Murao N Seino K Hayashi T Ikeda M Funayama E Furukawa H. Treg-enriched CD4+ T cells attenuate collagen synthesis in keloid fibroblasts. Exp Dermatol (2014) 23(4):266–71. doi: 10.1111/exd.12368 24617809

[68] Funayama E Chodon T Oyama A Sugihara T. Keratinocytes promote proliferation and inhibit apoptosis of the underlying fibroblasts: an important role in the pathogenesis of keloid. J Invest Dermatol (2003) 121(6):1326–31. doi: 10.1111/j.1523-1747.2003.12572.x 14675177

[69] Chipev CC Simman R Hatch G Katz AE Siegel DM Simon M. Myofibroblast phenotype and apoptosis in keloid and palmar fibroblasts *in vitro* . Cell Death Differ (2000) 7(2):166–76. doi: 10.1038/sj.cdd.4400605 10713731

[70] Arciniegas E Carrillo LM Rojas H Ramirez R Chopite M. Galectin-1 and galectin-3 and their potential binding partners in the dermal thickening of keloid tissues. Am J Dermatopathol (2019) 41(3):193–204. doi: 10.1097/DAD.0000000000001284 30801341

[71] Zhuang Z Li Y Wei X. The safety and efficacy of intralesional triamcinolone acetonide for keloids and hypertrophic scars: A systematic review and meta-analysis. Burns (2021) 47(5):987–98. doi: 10.1016/j.burns.2021.02.013 33814214

[72] Hochman B Locali RF Matsuoka PK Ferreira LM. Intralesional triamcinolone acetonide for keloid treatment: a systematic review. Aesthetic Plast Surg (2008) 32(4):705–9. doi: 10.1007/s00266-008-9152-8 18418647

[73] Nangole FW Ouyang K Anzala O Ogengo J Agak GW. Multiple cytokines elevated in patients with keloids: Is it an indication of auto-inflammatory disease? J Inflammation Res (2021), 14, 2465–70. doi: 10.2147/JIR.S312091 PMC8203597 34140794

[74] Hsu SY Liang SG Hsueh AJ. Characterization of two LGR genes homologous to gonadotropin and thyrotropin receptors with extracellular leucine-rich repeats and a G protein-coupled, seven-transmembrane region. Mol Endocrinol (1998) 12(12):1830–45. doi: 10.1210/mend.12.12.0211 9849958

[75] Barker N van Es JH Kuipers J Kujala P van den Born M Cozijnsen M. Identification of stem cells in small intestine and colon by marker gene Lgr5. Nature (2007) 449(7165):1003–7. doi: 10.1038/nature06196 17934449

[76] Kemper K Prasetyanti PR De Lau W Rodermond H Clevers H Medema JP. Monoclonal antibodies against Lgr5 identify human colorectal cancer stem cells. Stem Cells (2012) 30(11):2378–86. doi: 10.1002/stem.1233 22969042

[77] Liu XS Lin XK Mei Y Ahmad S Yan CX Jin HL. Regulatory T cells promote overexpression of Lgr5 on gastric cancer cells *via* TGF-beta1 and confer poor prognosis in gastric cancer. Front Immunol (2019) 101741:1741. doi: 10.3389/fimmu.2019.01741 PMC6682668 31417548

[78] Papadimitriou E Mikelis C Lampropoulou E Koutsioumpa M Theochari K Tsirmoula S. Roles of pleiotrophin in tumor growth and angiogenesis. Eur Cytokine Netw (2009) 20(4):180–90. doi: 10.1684/ecn.2009.0172 20167557

[79] Giddings BH Kwong SL Parikh-Patel A Bates JH Snipes KP. Going against the tide: increasing incidence of colorectal cancer among koreans, filipinos, and south asians in California, 1988-2007. Cancer Causes Control (2012) 23(5):691–702. doi: 10.1007/s10552-012-9937-6 22460700

[80] Mikelis C Koutsioumpa M Papadimitriou E. Pleiotrophin as a possible new target for angiogenesis-related diseases and cancer. Recent Pat Anticancer Drug Discovery (2007) 2(2):175–86. doi: 10.2174/157489207780832405 18221061

[81] Herradon G Ramos-Alvarez MP Gramage E. Connecting metainflammation and neuroinflammation through the PTN-MK-RPTPbeta/zeta axis: Relevance in therapeutic development. Front Pharmacol (2019) 10377:377. doi: 10.3389/fphar.2019.00377 PMC6474308 31031625

[82] Huang C Nie F Qin Z Li B Zhao X. A snapshot of gene expression signatures generated using microarray datasets associated with excessive scarring. Am J Dermatopathol (2013) 35(1):64–73. doi: 10.1097/DAD.0b013e31825ba13f 22785331

[83] Lee DH Jin CL Kim Y Shin MH Kim JE Kim M. Pleiotrophin is downregulated in human keloids. Arch Dermatol Res (2016) 308(8):585–91. doi: 10.1007/s00403-016-1678-z 27465069

[84] Arjunan S Gan SU Choolani M Raj V Lim J Biswas A. Inhibition of growth of Asian keloid cells with human umbilical cord wharton's jelly stem cell-conditioned medium. Stem Cell Res Ther (2020) 11(1):78. doi: 10.1186/s13287-020-01609-7 32085797 PMC7035736

[85] Bolos V Grego-Bessa J de la Pompa JL. Notch signaling in development and cancer. Endocr Rev (2007) 28(3):339–63. doi: 10.1210/er.2006-0046 17409286

[86] Chigurupati S Arumugam TV Son TG Lathia JD Jameel S Mughal MR. Involvement of notch signaling in wound healing. PloS One (2007) 2(11):e1167. doi: 10.1371/journal.pone.0001167 18000539 PMC2048753

[87] Allenspach EJ Maillard I Aster JC Pear WS. Notch signaling in cancer. Cancer Biol Ther (2002) 1(5):466–76. doi: 10.4161/cbt.1.5.159 12496471

[88] Syed F Bayat A. Notch signaling pathway in keloid disease: enhanced fibroblast activity in a jagged-1 peptide-dependent manner in lesional vs. extralesional fibroblasts. Wound Repair Regener (2012) 20(5):688–706. doi: 10.1111/j.1524-475X.2012.00823.x 22985040

[89] Russell SB Russell JD Trupin KM Gayden AE Opalenik SR Nanney LB. Epigenetically altered wound healing in keloid fibroblasts. J Invest Dermatol (2010) 130(10):2489–96. doi: 10.1038/jid.2010.162 PMC2939920 20555348

[90] Wang H Quan L Liang J Shi J Qiu T Zhang Y. Gene expression profiling analysis of keloids with and without hydrocortisone treatment. Exp Ther Med (2017) 14(6):5283–8. doi: 10.3892/etm.2017.5263 PMC5740600 29285054

[91] Condorelli AG Logli E Cianfarani F Teson M Diociaiuti A El Hachem M. MicroRNA-145-5p regulates fibrotic features of recessive dystrophic epidermolysis bullosa skin fibroblasts. Br J Dermatol (2019) 181(5):1017–27. doi: 10.1111/bjd.17840 30816994

[92] Wang K Zhang Y Li X Chen L Wang H Wu J. Characterization of the kremen-binding site on Dkk1 and elucidation of the role of kremen in dkk-mediated wnt antagonism. J Biol Chem (2008) 283(34):23371–5. doi: 10.1074/jbc.M802376200 PMC2516984 18502762

[93] Grotewold L Theil T Ruther U. Expression pattern of dkk-1 during mouse limb development. Mech Dev (1999) 89(1-2):151–3. doi: 10.1016/s0925-4773(99)00194-x 10559490

[94] Ueland T Astrup E Otterdal K Lekva T Janardhanan J Prakash JAJ. Secreted wnt antagonists in scrub typhus. PloS Negl Trop Dis (2021) 15(4):e0009185. doi: 10.1371/journal.pntd.0009185 33914733 PMC8112706

[95] Aslam N Abusharieh E Abuarqoub D Ali D Al-Hattab D Wehaibi S. Anti-oncogenic activities exhibited by paracrine factors of MSCs can be mediated by modulation of KITLG and DKK1 genes in glioma SCs *in vitro* . Mol Ther Oncolytics (2021), 20, 147–65. doi: 10.1016/j.omto.2020.11.005 PMC7851499 33575478

[96] Sui Q Liu D Jiang W Tang J Kong L Han K. Dickkopf 1 impairs the tumor response to PD-1 blockade by inactivating CD8+ T cells in deficient mismatch repair colorectal cancer. J Immunother Cancer (2021) 9(3). doi: 10.1136/jitc-2020-001498 PMC8009229 33782107

[97] D'Amico L Mahajan S Capietto AH Yang Z Zamani A Ricci B. Dickkopf-related protein 1 (Dkk1) regulates the accumulation and function of myeloid derived suppressor cells in cancer. J Exp Med (2016) 213(5):827–40. doi: 10.1084/jem.20150950 PMC4854727 27045006

[98] Kikuchi A Matsumoto S Sada R. Dickkopf signaling, beyond wnt-mediated biology. Semin Cell Dev Biol (2022), 125, 55–65. doi: 10.1016/j.semcdb.2021.11.003 34801396

[99] Aguilera O Fraga MF Ballestar E Paz MF Herranz M Espada J. Epigenetic inactivation of the wnt antagonist DICKKOPF-1 (DKK-1) gene in human colorectal cancer. Oncogene (2006) 25(29):4116–21. doi: 10.1038/sj.onc.1209439 16491118

[100] Maehata T Taniguchi H Yamamoto H Nosho K Adachi Y Miyamoto N. Transcriptional silencing of dickkopf gene family by CpG island hypermethylation in human gastrointestinal cancer. World J Gastroenterol (2008) 14(17):2702–14. doi: 10.3748/wjg.14.2702 PMC2709050 18461655

[101] Chu HY Chen Z Wang L Zhang ZK Tan X Liu S. Dickkopf-1: A promising target for cancer immunotherapy. Front Immunol (2021), 12, 658097. doi: 10.3389/fimmu.2021.658097 34093545 PMC8174842

[102] Jin Q Gui L Niu F Yu B Lauda N Liu J. Macrophages in keloid are potent at promoting the differentiation and function of regulatory T cells. Exp Cell Res (2018) 362(2):472–6. doi: 10.1016/j.yexcr.2017.12.011 29253537

[103] Chen Z Zhou L Won T Gao Z Wu X Lu L. Characterization of CD45RO(+) memory T lymphocytes in keloid disease. Br J Dermatol (2018) 178(4):940–50. doi: 10.1111/bjd.16173 29194570

